# Effect of Jet Impingement Velocity and Angle on CO_2_ Erosion–Corrosion with and without Sand for API 5L-X65 Carbon Steel

**DOI:** 10.3390/ma13092198

**Published:** 2020-05-11

**Authors:** Ihsan Ulhaq Toor, Zakariya Alashwan, Hassan Mohamed Badr, Rached Ben-Mansour, Siamack A. Shirazi

**Affiliations:** 1 Department of Mechanical Engineering, King Fahd University of Petroleum & Minerals (KFUPM), Dhahran 31261, Saudi Arabia; g200628940@kfupm.edu.sa (Z.A.); badrhm@kfupm.edu.sa (H.M.B.); rmansour@kfupm.edu.sa (R.B.-M.); 2 Ju’aymah NGL Plant, Saudi Aramco, Ras Tanura 31311, Saudi Arabia; 3 Erosion/Corrosion Research Center, Department of Mechanical Engineering, The University of Tulsa, Tulsa, OK 74104, USA; siamack-shirazi@utulsa.edu

**Keywords:** CO_2_ erosion–corrosion, flow loop, API 5L X-65, surface roughness, XRD, EDS, SEM

## Abstract

Most oil and gas production wells have plenty of corrosive species present along with solid particles. In such production environments, CO_2_ gas can dissolve in free phase water and form carbonic acid (H_2_CO_3_). This carbonic acid, along with fluid flow and with/without solid particles (sand or other entrained particles), can result in unpredictable severe localized CO_2_ corrosion and/or erosion–corrosion (EC). So, in this work, the CO_2_ EC performance of API 5L X-65 carbon steel, a commonly used material in many oil and gas piping infrastructure, was investigated. A recirculating flow loop was used to perform these studies at three different CO_2_ concentrations (pH values of 4.5, 5.0, and 5.5), two impingement velocities (8 and 16 m/s), three impingement angles (15°, 45°, and 90°), and with/without 2000 ppm sand particles for a duration of 3 h in 0.2 M NaCl solution at room temperature. Corrosion products were characterized using FE-SEM, EDS, and XRD. The CO_2_ EC rates were found to decrease with an increase in the pH value due to the increased availability of H^+^ ions. The highest CO_2_ erosion–corrosion rates were observed at a 45° impingement angle in the presence of solid particles under all conditions. It was also observed that a change in pH value influenced the morphology and corrosion resistance of the corrosion scales.

## 1. Introduction

In most oil and gas production environments, it is very difficult to avoid the presence of corrosive species and solid particles. Commonly used sand screens in oil and gas production wells cannot filter sand particles below 50 µm [1,2,3]. The flowing fluids also carry CO_2_ gas produced by most of the hydrocarbon wells and/or extracted by some processing units. In addition, CO_2_ gas is also injected to lift up the hydrocarbon and to increase the hydrocarbon production from the wells [4]. Carbon dioxide gas can dissolve in free phase water and form carbonic acid (CO_2_ + H_2_O ⇔ H_2_CO_3_). Formation of carbonic acid (H_2_CO_3_) in free phase water brings down the solution pH and can result in severe localized CO_2_ erosion–corrosion [5]. Most facilities dealing with CO_2_ gas are experiencing CO_2_ erosion–corrosion issues in their assets, mostly made of carbon steel and low alloy steels [4,6,7].

Various parameters may affect CO_2_ erosion–corrosion rates such as CO_2_ partial pressure (pCO_2_), solution pH, flow velocity, temperature, flow structure (impingement angle), and solid particles size/concentration [4,5,6,8]. It is well known that an increase in CO_2_ partial pressure (pCO_2_) will lower the solution pH due to the formation of carbonic acid. The relationship between pCO_2_ and solution pH is well documented in many references [5,9,10]. Lowering the solution pH will result in higher CO_2_ erosion–corrosion, as the hydrogen reduction rate will be increased [8,11]. On the other hand, fluid velocity can significantly affect CO_2_ erosion–corrosion rates, not only by increasing the turbulent intensities, but also by increasing the impact velocity of solid particles, and that can be described as CO_2_ flow accelerated corrosion or erosion–corrosion. The turbulent flow along with increased solid particle velocity will increase the mass transfer of the species to and away from the metal surface. This will induce more stresses and will tend to break and/or remove passive films; this will ultimately increase the CO_2_ erosion–corrosion. The fluid flow can also affect the erosion–corrosion rate by particle impingement on the metal surface. This will erode the material/passive film away and expose the bare metal which will enhance the erosion–corrosion. Increasing the fluid velocity along with the availability of sand particles will increase the loading and stresses of sand particles hitting the metal surface and thus the CO_2_ erosion–corrosion rate will increase [6,9,12,13,14,15].

There is some published literature discussing the effect of impinging jets (mostly normal impingement angles) on the erosion–corrosion behavior of carbon steels in different environments [16,17]. It is worth mentioning though that the impingement angle and velocity are important experimental parameters in the study of erosion–corrosion, as they can significantly affect the deterioration of target material. On the other hand, the ductility of the target material and the availability of solid particles in the fluid can play a significant role in CO_2_ erosion–corrosion [18]. The effect of two different types of stresses (i.e., normal and shear) resulting from fluid impingement on a metal surface has been discussed by different researchers [8,15,18]. The normal stress, resulting from a fluid jet acting normally on a metal surface, and lateral shear stress results from the flowing fluid force parallel to the metal surface. Varying the impingement angle and velocity will vary the distribution of normal and shear stresses over the metal surface [8].

A synergistic effect of CO_2_ erosion–corrosion was observed by several researchers [9,19,20,21]. This synergistic effect can result in much more deterioration than caused by the sum of individual corrosion reactions (electrochemical) and erosion (mechanical) mechanisms. It was observed that erosion and corrosion activities enhance each other which makes it quite complicated to predict the “equipment life” accurately [21]. To overcome CO_2_ erosion–corrosion issues, corrosion modeling to predict asset life under CO_2_ erosion–corrosion can be developed with the use of numerical correlations. For this reason, a comprehensive and reliable CO_2_ erosion–corrosion database is required to build these numerical correlations. Such comprehensive databases are rarely reported in the published literature and can be produced by performing experiments using flow loops. Flow loops are not often used in laboratories because of their high cost of construction, maintenance, and large space requirements in the laboratories.

It is obvious from the above discussion that, in general, CO_2_ erosion–corrosion investigations are carried out using different additives to control the required solution pH. There are not many reported results on the effects of flow velocities, pH and angles on CO_2_ erosion–corrosion in recirculating flow loops. Therefore, the objective of this work was to study the CO_2_ erosion–corrosion behavior of API 5L-X65 carbon steel in varying conditions of pH, flow velocity, and impingement angles while using a recirculation flow loop to simulate a real-time environment. These data will assist in creating a CO_2_ erosion–corrosion database which will be used to develop CO_2_ erosion–corrosion prediction models.

## 2. Materials and Methods

### 2.1. Equipment

**Figure 1:**
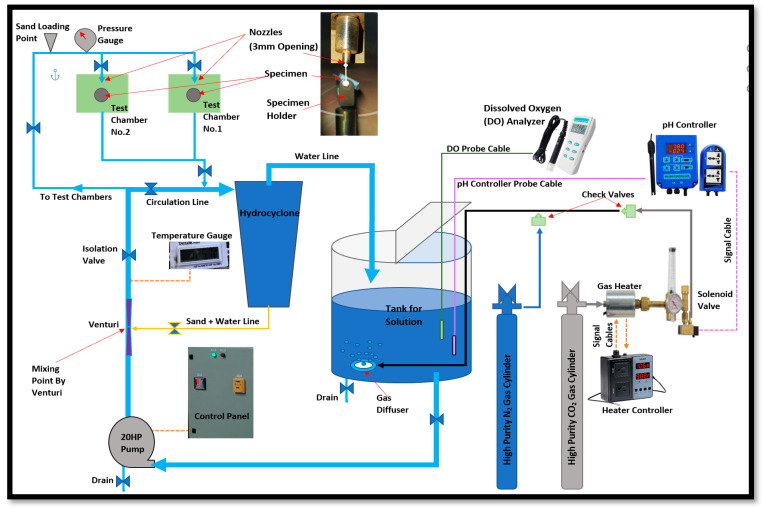
Sketch for CO_2_ erosion–corrosion Flow Loop.

An impingement flow loop was used to perform CO_2_ erosion–corrosion testing as shown in Figure 1. This stainless-steel flow loop was equipped with a centrifugal pump which was connected to piping, a tank, two testing chambers, a hydrocyclone, and an injector. In the test chamber, the impingement angle could be varied from 15° up to 90°. The pump was equipped with a speed controller to adjust the fluid velocity. The flow loop was also equipped with a hydrocyclone and an injector to protect the pump from erosion–corrosion, and they controlled the injection and separation of the sand, respectively. In addition, modifications were made in the flow loop for CO_2_ erosion–corrosion experiments, by connecting a high purity N_2_ and CO_2_ gas cylinders to the tank along with a nano gas diffuser (suspended inside the tank for better purging). Moreover, devices such as a pH controller, solenoid valve, gas check valves, gas heater, heat controller, and dissolved oxygen analyzer were used to simulate and control the testing conditions. The pH controller continuously monitored the solution pH and subsequently kept sending signals to the solenoid valve to open or close for CO_2_ purging based on previous pH settings. The relationship between dissolved CO_2_ concentrations and solution pH is well documented in many references [5,9,10]. With the help of the pH controller, the dissolved CO_2_ concentration was controlled by closely controlling the CO_2_ gas purging rate. This ultimately helped to maintain the required pH by controlling dissolved CO_2_ concentration. The pH controller was provided by Gain Express Holding Ltd., and this pH controller has a measurement range of 0–14.

### 2.2. Test Specimens

CO_2_ erosion–corrosion testing was conducted on API5L X65 Carbon Steel specimens. Elemental composition was conducted three times by a SPECROMAXx metal analyzer, and it showed the elemental composition as given here: 0.162 wt % C; 1.27wt % Mn; 0.0082 wt % P; 0.0068 wt % S; 0.0010 wt % Ti; 0.0106 wt % V; 0.0340 wt % Nb–Fe balance. In addition, Vickers hardness testing was performed by a CSM Micro Combi Hardness Tester with diamond indenter under 500 g-force “gf” (4.903325 N load). The hardness test was repeated ten times and an average value of 177.1 HV was obtained.

**Figure 2:**
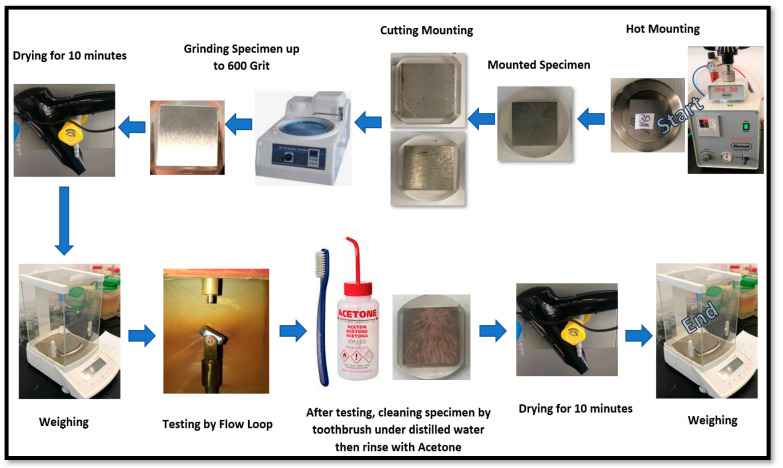
Testing specimens’ preparation steps before and after CO_2_ erosion–corrosion.

Square specimens with dimensions of 20 mm × 20 mm × 5 mm (thickness) were prepared in the machine shop from 8 inch pipe (OD 8.66 inch) of API 5L X65 Carbon Steel with a 0.5 inch thickness. Figure 2 shows the specimen preparation steps before and after CO_2_ erosion–corrosion.

### 2.3. Test Solution

A 0.2 M NaCl testing solution was made to simulate the industrial corrosive conditions by dissolving 1606.52 g of ACS grade NaCl in 137.45 L of tap water (drinking sweet water). An analysis of used tap water was carried out, and it showed a total dissolved solids (TDS) content equal to 164.2 ppm, 7.5 pH, and chloride ion content of 140 ppm. Then, the solution was purged with high-purity N_2_ gas (99.999%) at 25 psi for 30 min to remove dissolved oxygen. Less than 40 ppb of dissolved oxygen was observed after 30 min of N_2_ purging. After that, solution was purged with high-purity CO_2_ gas (99.99%) until set pH value was achieved (pH 4.5, 5.0 and 5.5). For CO_2_ erosion–corrosion experiments with sand, the solution was mixed with 2000 ppm of sand particles (99.54% SiO_2_) having an average particle size of 190 µm. To avoid oxygen ingress into the system, the sand was loaded into the flow loop from the sand loading point before N_2_ purging. The closure of the valves located before and after the specimen chambers was ensured during the sand loading and gas (N_2_, CO_2_) purging.

### 2.4. Testing Procedure

The CO_2_ erosion–corrosion experiments were conducted by utilizing a flow loop which was made of stainless-steel grade (316 L) and capable of varying the impingement velocity and angle. The experiments were performed at room temperature and in accordance with ASTM-G-73-98 [22]. The tank in Figure 1 was filled with 137.45 L of 0.2 M NaCl solution and purged first with N_2_ gas and later with CO_2_ gas as specified previously. The CO_2_ concentration was controlled with the help of a pH controller to maintain three different pH values (4.5, 5.0, and 5.5) in different experiments. For CO_2_ erosion–corrosion experiments with sand, the solution was mixed with 2000 ppm of sand particles.

Test specimens were machined, hot mounted, and ground up to 600 grit emery paper. The specimens were subsequently dried for 10 min and weighed using a digital balance up to 0.01 mg. Prepared specimens were fixed (one specimen at each testing chamber) inside the testing chambers. Three impingement angles—15°, 45,° and 90°—were used along with two different fluid impingement velocities of 8 and 16 m/s. Once the solution was ready, the experiment was started by closing the circulation line valve and opening the valves before and after the test chambers. Moreover, trapped air was vented from the test chambers utilizing venting bolts. The solution was purged with N_2_ to remove the oxygen and a concentration of 40 ppb was maintained before the start of each experiment.

After 3 h of the CO_2_ erosion–corrosion experiment, specimens were taken out of the test chamber, rinsed with distilled water, cleaned with soft toothbrush and subsequently rinsed with acetone. After that, the specimens were dried for 10 min and then the final weights were measured using a digital balance up to 0.01 mg. This weight loss was used to calculate the corrosion rate (mm/y) under that particular experimental condition. Each experiment was repeated twice by changing one parameter condition (pH, impingement velocity, angle, with and without sand).

### 2.5. Surface and Corrosion Scales Characterization

Cross-sections of corrosion scales were observed using field emission scanning electron microscopy (FE-SEM). Also, the corrosion scales’ compositions were identified with X-ray diffraction spectroscopy (XRD) and energy-dispersive X-ray spectroscopy (EDS).

## 3. Results and Discussion

### 3.1. Visual Observation

**Figure 3:**
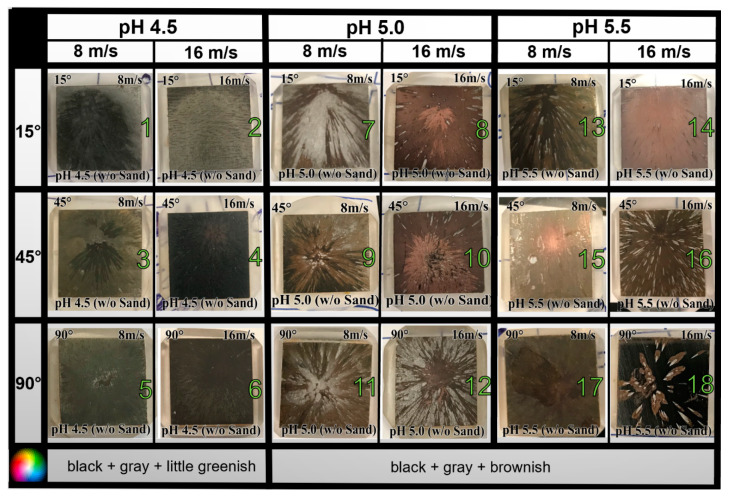
Pictures of API 5L X65 specimens after testing in CO_2_ flow accelerated corrosion (FAC) at three pH levels (4.5, 5.0, 5.5), three angles (15°, 45°, 90°), two velocities (8, 16 m/s), and at room temperature.

**Figure 4:**
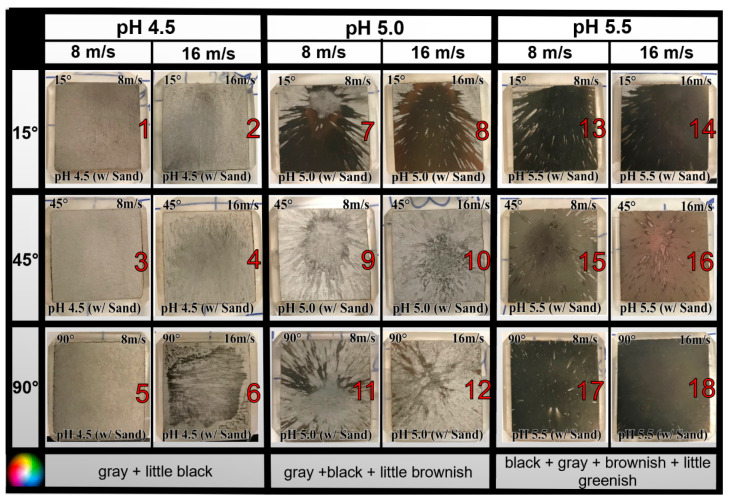
Pictures of API 5L X65 specimens after testing in CO_2_ erosion–corrosion (EC) with sand at three pH levels (4.5, 5.0, 5.5), three angles (15°, 45°, 90°), two velocities (8, 16 m/s), and at room temperature.

In order to observe the degradation (in terms of coloring and corrosion pattern), pictures were taken for all the tested specimens soon after the test (after surface was cleaned). This pictorial evidence was categorically arranged in order to have a clear visual comparison of the effects of solution pH, impingement angle, velocity, and sand particles [1,23]. Impingement spots surrounded by scars were observed in most of the tested specimens as shown in Table 1. Figure 3 and Figure 4 shows the pictures of tested specimens without and with sand particles, respectively. The dark coloring on the specimens is the result of corrosion products as described in Section 3.2.

### 3.2. Corrosion Scales Phase Identification by XRD

**Figure 5:**
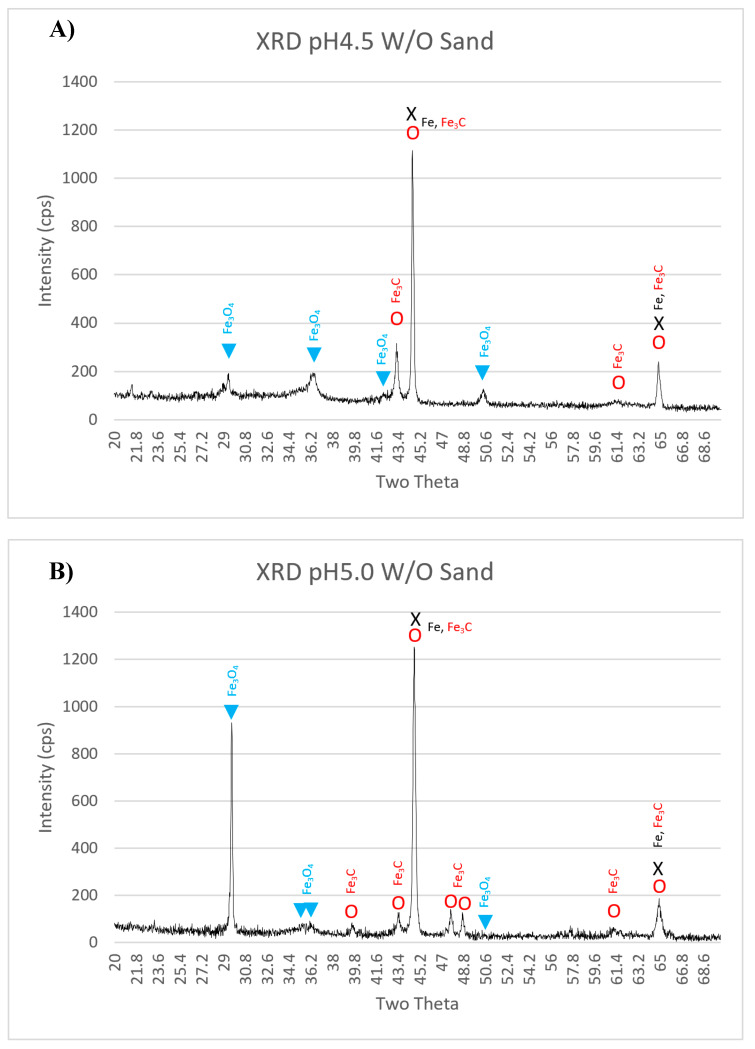

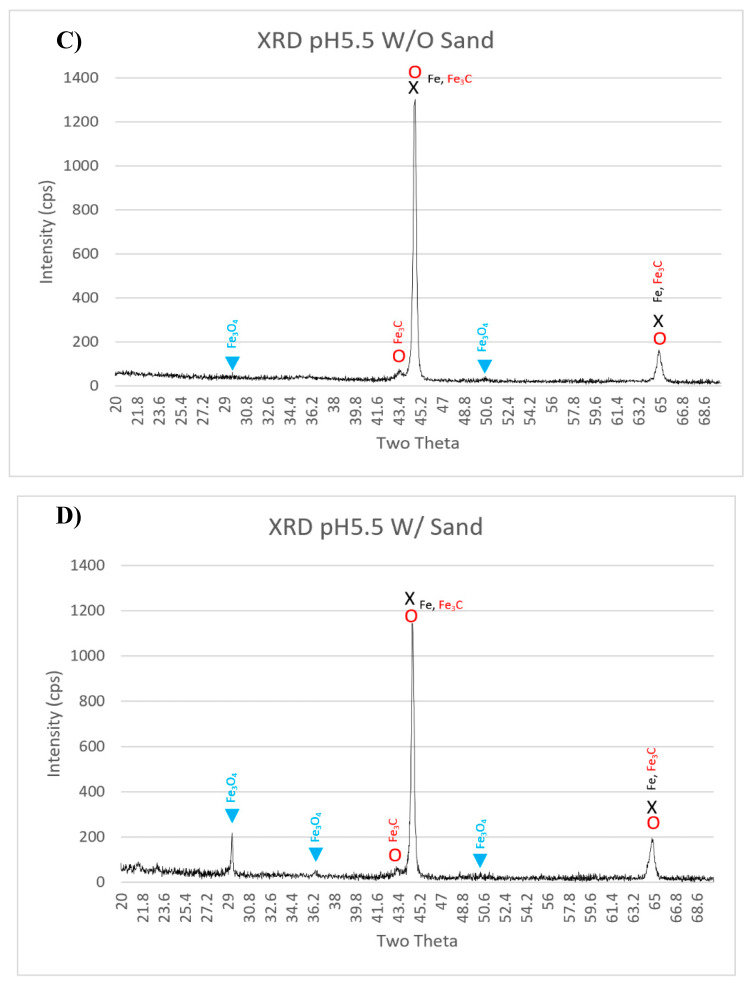
XRD peaks for API 5L X65 tested in CO_2_ IC API 5l X65 tested in CO_2_ EC at impingement angle 90°, room temperature, impingement velocity 8 m/s: (**A**) pH 4.5 without sand, (**B**) pH 5.0 without sand, (**C**) pH 5.5 without sand, and (**D**) pH 5.5 with sand.

A Rigaku MiniFlexII XRD (Tokyo, Japan) was used to identify the corrosion scales found on the tested specimens. A Cu (Kα) radiation was used to obtain the radiation spectrum with a spot width of 12 mm, over a 2θ range (20–70°) and with a step size of 0.02°/min at 20 kv. Figure 5 shows XRD peaks for different phases observed on specimens with different pH levels, fixed impingement velocity of 8 m/s, and an impingement angle of 90°. All the specimens exhibited three different phases which were iron (Fe) [23], cementite (Fe_3_C) [24,25], and magnetite (Fe_3_O_4_) [26]. The intensity of the peaks was less in the case of specimens tested at pH 5.5 with and without sand, probably because of the less material degradation at higher pH values. Moreover, for the specimen tested at pH 5.5 with sand, the sand particles were continuously eroding the corrosion products, which resulted in thinner corrosion scale.

**Figure 6:**
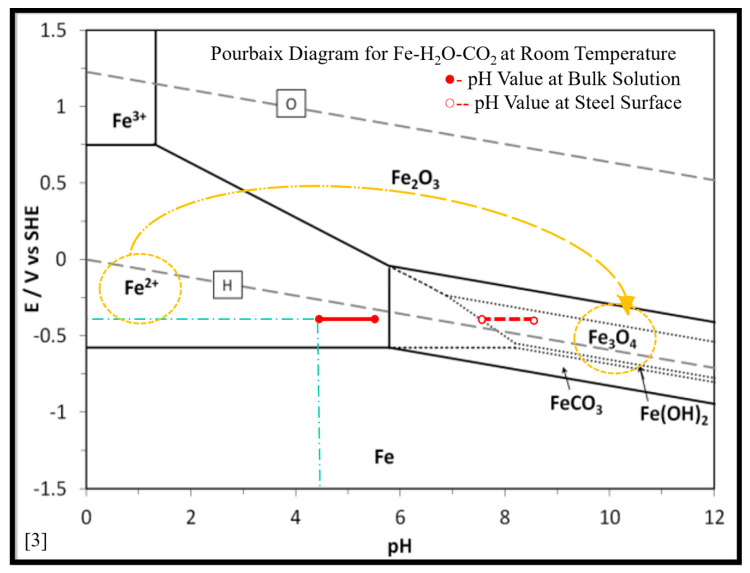
Pourbaix Diagram for Fe–H_2_O–CO_2_ at room temperature, cfe^2+^ = 10 ppm, cfe^3+^ = 10 ppm, hydrogen partial pressure 1 bar, oxygen partial pressure 1 bar, carbon dioxide partial pressure 1 bar (symbols: ●- pH value at bulk solution, ○-- pH value at steel surface) [26].

**Figure 7:**
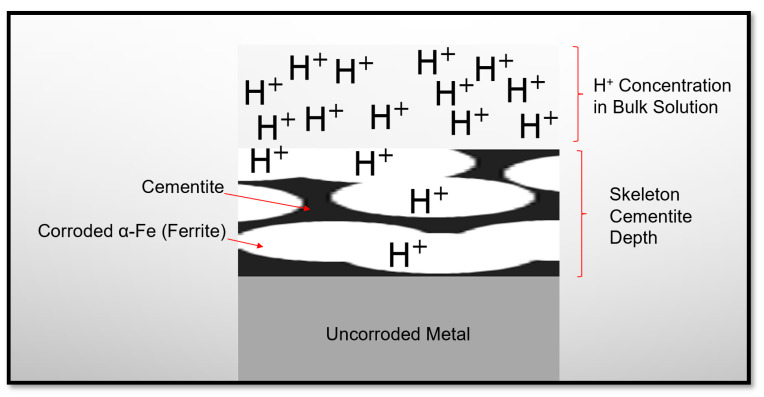
Schematic for skeleton cementite as a result of α-Fe phase corrosion.

It was reported elsewhere [27] that the corrosion rate of carbon steel was almost constant when experiments were performed under the conditions (temperature = 50 °C, pH = 6.6, P_CO2_ = 0.54 bar, c_Fe2+_ = 250 ppm, v = 1 m/s, t = 30 h), as there was no iron carbonate film formation. However, iron carbonate film was formed after 0.5, 5, and 15 h, respectively, when temperature was increased to 55 °C, 65 °C and 80 °C, respectively. This shows a strong relationship between solution temperature and FeCO_3_ corrosion product formation in CO_2_ aqueous environments. It was also reported by different researchers [25] that the formation of FeCO_3_ corrosion products is less likely at low temperature (≤40 °C), as FeCO_3_ will be unstable under this temperature. Benezeth et al. [28] reported the formation of FeCO_3_, when the experiments were conducted for 18 days in a hydrogen-electrode concentration cell at 25 °C with injected PCO_2_ of 4 bar. They also reported the presence of Fe_3_O_4_ as per their XRD results which shows the possibility of oxides scale formation along with iron carbonate scales under the tested conditions. The experimental results presented in this work show the formation of Fe_3_O_4_ instead of FeCO_3_ when the experiments were performed at 25 °C for 3 h. These oxide scales may have formed due to the slight oxygen ingress into the tank solution during the experiments, as the setup was completely airtight. The practical corrosion potential for API 5L X65 steel in a CO_2_ EC environment is approximately −0.35 Vvs SHE [8]. It is clear from the Pourbaix diagram (Figure 6) of Fe–H_2_O–CO_2_ at room temperature (25 °C) [29], that Fe^2+^ ions (corrosion) will be stable at the tested pH values (4.5, 5.0, and 5.5). As carbon steel has α-Fe (ferrite) and cementite phase, so at the tested pH values, the α-Fe phase will disassociate to Fe^2+^ ions (corrosion), leaving behind a skeleton (empty) of cementite (Fe_3_C) phase which is not corroded [6,30,31]. Mora-Mendoza and Turgoose [31] discussed in their work that the thickness of uncorroded cementite (Fe_3_C) phase in a CO_2_ containing environment will become thicker with time and may reach up to 75 µm in specific conditions. Saeid et al. [32] studied the steel surface’s pH in a CO_2_ corrosion environment and concluded that under the stagnation condition, the steel surface pH will be higher than that of the bulk solution pH by about three pH values. Due to the fact of this, in the case of skeleton Fe_3_C film formation, this film will limit H^+^ ions exchange between bulk solution and inside the Fe_3_C film and will lead to an increase of solution pH value inside the Fe_3_C film as shown schematically in Figure 7 [28]. It is estimated based on the work carried by Saeid et al. [32] that pH will be increased at least by a value three, and this estimation was used to explain Figure 7. Moreover, it is discussed by some researchers that precipitation of corrosion products inside Fe_3_C film will make it more resistance to turbulence flow [31]. From the Pourbaix diagram of Fe–H_2_O–CO_2_ at room temperature (Figure 6), it is clear that by increasing the pH value of solution inside the Fe_3_C film will lead to the formation of Fe_3_O_4_ (magnetite) phase, as it will be more stable than Fe^2+^ ions [29]. Formation of Fe_3_C and Fe_3_O_4_ phases were confirmed by XRD peaks (Figure 5) obtained for the tested specimens.

### 3.3. Cross-Section Characterization of Corrosion Scales

#### 3.3.1. FE-SEM Observations

**Figure 8:**
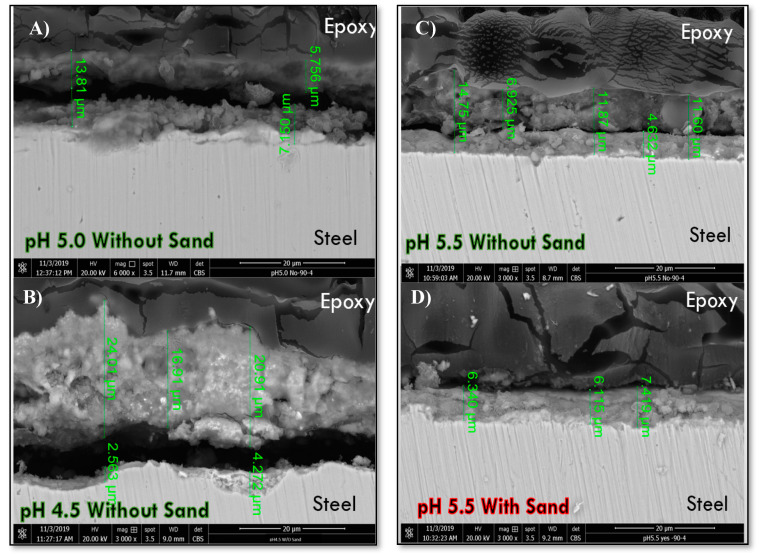
Cross-sectional FE-SEM backscattered images for API 5L X65 tested in CO_2_ EC at an impingement angle of 90°, room Temperature, impingement velocity of 8 m/s: (**A**) pH 5.0 without sand, (**B**) pH 4.5 without sand, (**C**) pH 5.5 without sand, and (**D**) pH 5.5 with sand.

**Figure 9:**
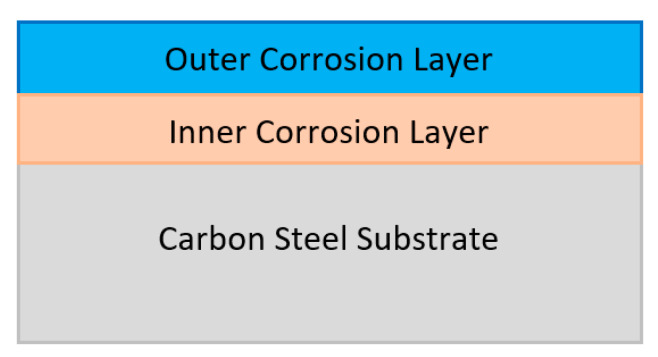
Schematic of formed corrosion products.

Cross-sectional corrosion scale thickness of some selected specimens was measured by FE-SEM, and the results are shown in Figure 8. Corrosion scale thickness measurements were made at various locations on the same specimen, and an average thickness value is reported here. For the cases without sand particles, the corrosion scale thickness was increased with a decrease in pH value, and the highest scale thickness (~23 µm) was observed at pH 4.5. Intermediate corrosion scale thicknesses was observed for pH 5.5 (~12 µm) and pH 5.0 (~13 µm), respectively. However, the corrosion scale, on the specimen tested at pH 5.5 with sand, showed the lowest thickness (~7 µm) due to the sand erosion. Moreover, two layers of corrosion scales were observed on top of the steel surfaces tested at pH 4.5, 5.0, and 5.5 without sand (Figure 9). Whereas, only one adhered layer was found for the specimen tested at pH 5.5 in the presence of sand particles. The inner corrosion layer formed on top of the steel surface seems to be intact and adherent to the steel surface. Tonje et al. [30] mentioned that only corrosion products (other than or mixed with Fe_3_C) formed directly on top of steel surface can be protective in a CO_2_ containing environment. In addition, no outer corrosion layer was observed on the top of the adherent inner layer for specimens tested at pH 5.5 with sand. This means that sand eroded the outer corrosion layer or prevented its formation because it was loose and poorly attached to the inner layer. It is worth mentioning that the thickness of the inner adherent corrosion layer was found to increase with an increase in pH, and the thickness of the outer/non-adherent corrosion layer decreased with an increase in pH value. Also, a gap was noticed between the two corrosion layers which resulted from smooth polishing and a non-adherent outer layer. In addition, less porosities were observed in the inner corrosion layers formed at pH 5.5 with and without sand (Figure 8), while more porosities were clearly shown in the inner corrosion layer formed at pH 5.0 without sand (Figure 8) which means that more corrosion resistant film was formed at pH 5.5 (higher pH values).

Mora-Mendoza and Turgoose [31] studied the CO_2_ corrosion phenomenon in the absence of sand particles. They reported in their work that the Fe_3_C film was intact and was not remove easily by turbulence flow induced at 1000 rpm. There are other researchers who have reported [8,11,29] that the CO_2_ EC rate increases as the solution pH decreases because of the formation of carbonic acid (H_2_CO_3_) at lower pH values. So, for the cases without sand particles, a thicker Fe_3_C film was expected to form on the top of the carbon steel surface in solutions with lower pH values, which is in compliance with the FE-SEM results (Figure 8A–C). It was shown by the FE-SEM results (Figure 8) that thicker and adherent inner corrosion scales were formed with an increase in solution pH. This is owing to the fact that a little pH variation will be enough to reach a thermodynamically stable condition for Fe_3_O_4_ formation.

#### 3.3.2. Elemental Analysis by EDS

**Figure 10:**
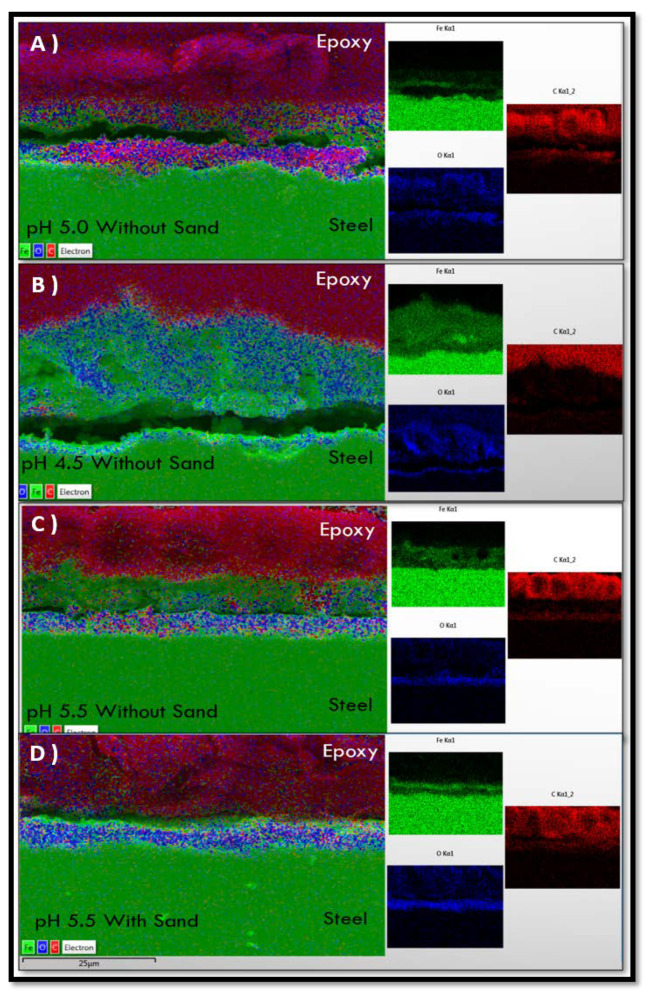
Elemental analysis and distribution for API 5L X65 tested in CO_2_ EC at impingement angle 90°, room temperature, impingement velocity of 8 m/s: (**A**) pH 5.0 without sand, (**B**) pH 4.5 without sand, (**C**) pH 5.5 without sand, and (**D**) pH 5.5 with sand.

The EDS elemental analysis of the corrosion scales showed the elemental distribution of the scales formed on carbon steel surface. Elemental mapping was obtained using a 3.5 nm scanning spot size. Figure 10 shows elemental composition and distribution of element inside the corrosion scales for different pH values and at an impingement angle of 90°. The major elements inside the corrosion products were iron (Fe), carbon (C), and oxygen (O) which confirmed the availability of cementite and magnetite phases in the formed corrosion products.

### 3.4. Effect of Varying Parameters on CO_2_ Erosion–corrosion Rates

**Figure 11:**
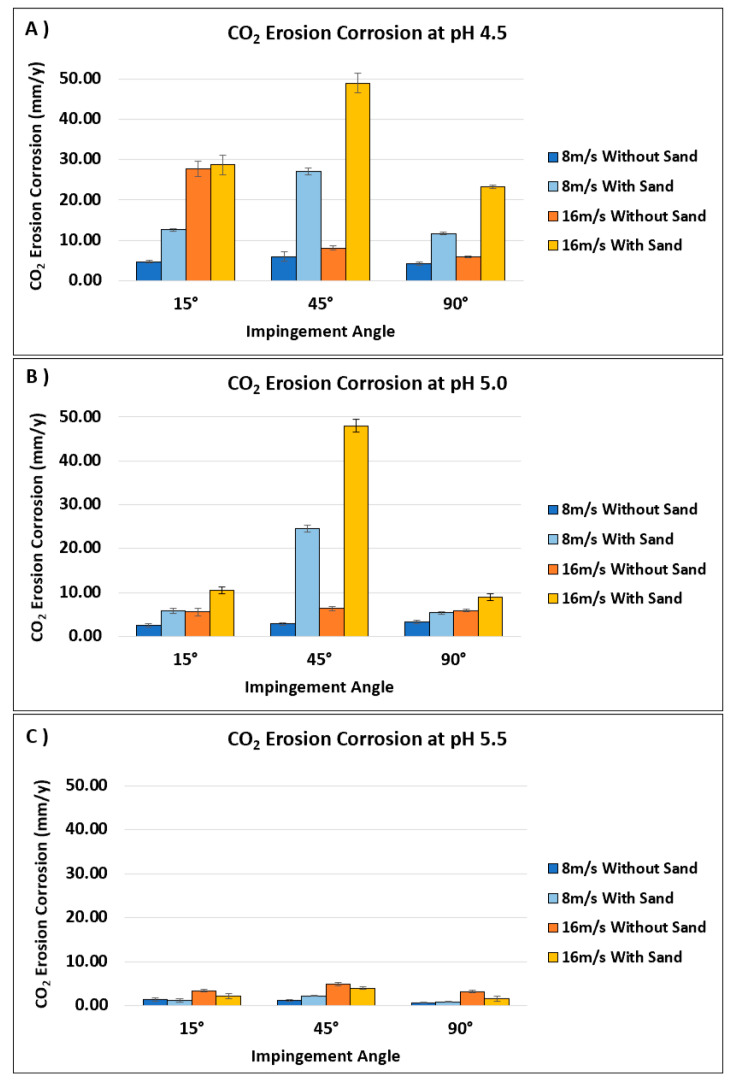
Performance of CO_2_ erosion–corrosion rates with varying impingement angles (15°, 45°, and 90°), impingement velocities (8 and 16 m/s), availability of sand particles (2000 ppm), and pH values: (**A**) pH 4.5, (**B**) pH 5.0, (**C**) pH 5.5.

Figure 11 shows the performance of CO_2_ erosion–corrosion rates with varying impingement angle (15°, 45°, and 90°), impingement velocity (8 and 16 m/s), pH value (4.5, 5.0, and 5.5), and availability of sand particles.

#### 3.4.1. Effect of CO_2_ Concentration Represented in pH Value

Dissolved CO_2_ gas concentration in the solution at atmospheric pressure can be controlled by purging CO_2_ gas into the solution. Carbonic acid (H_2_CO_3_) forms when solution is purged with CO_2_ gas. More purging of CO_2_ gas into the solution will result in more H_2_CO_3_ which will further dissociate to some degree and release H^+^ ions in the solution [9,30]. Availability of more H^+^ ions will decrease the pH value of the solution and make it more acidic. For this reason, in our experiments CO_2_ gas concentration was controlled by controlling the solution pH to specified pH values (4.5, 5.0, and 5.5), which complies with other reported literature [5,9,10]. Results of Figure 11 show that the CO_2_ EC rate decreased with an increase in pH value, as less CO_2_ gas was dissolved in the solution. These results are in agreement with what was discussed in Section 3.3.1.—that pH value influences the morphology of corrosion scales, i.e., their corrosion resistance [8,11,33].

#### 3.4.2. Effect of Impingement Angle

Cheng and Zhang [8] mentioned that localized stresses acting on the metal surface are responsible for plastic deformation owing to jet fluid. Some other researchers [8,18] reported that “normal and shear stresses” are mainly responsible for carbon steel erosion–corrosion. Varying the impingement angle and velocity will vary the distribution of normal and shear stresses over the metal surface [8,15,18]. Normal stress will be smaller at low impingement angles and vice versa for shear stress [18]. It was found that the CO_2_ EC rates were the highest at an impingement angle of 45° (Figure 11). The highest CO_2_ EC rate at 45° was probably due to the balance between normal and shear stresses which will tend to remove the corrosion products much deeper than at 15° [15,18]. However, different behavior was observed at pH 4.5 (without sand) at an impingement velocity of 16 m/s. At pH 4.5 (without sand), at an impingement velocity 16 m/s, the highest CO_2_ EC rate was observed at low impingement angle (15°). This behavior could be the result of a loosely attached thick outer corrosion layer formation at pH 4.5 in the absence of sand particles and which was easily thinned at a low impingement angle. Making the outer corrosion layer thinner will affect the stability of adhered inner corrosion layer and thus provide less of a way for corrosive species to pass through.

#### 3.4.3. Effect of Impingement Velocity

As per Figure 11, the CO_2_ EC rates were found to increase with an increase in impingement velocity. The highest CO_2_ EC rate was observed for the specimen tested at pH 4.5 (with sand particles) at an impingement velocity of 16 m/s and impingement angle of 45°. While the lowest CO_2_ EC rate was found for the specimen tested at pH 5.5 (without sand) at an impingement velocity of 8 m/s and impingement angle of 90°.

The increase in the EC rate at higher impingement velocity was mainly due to the increased loading of corrosive species (mass transfer) to and away from the target surface. It is discussed elsewhere by Toor et al. [18] that an increase in mass transfer will increase the chemical dissolution which will result in higher corrosion rates. It was discussed in previous sections that the CO_2_ EC rate increased with a decrease in pH value due to the availability of more corrosive H^+^ ions in the solution. Therefore, a higher impingement velocity (16 m/s) resulted in a higher chemical dissolution, especially at lower pH, i.e., in the presence of more corrosive H^+^ ions (pH 4.5) and that ultimately increased the CO_2_ EC. Along with these factors, the applied stresses on the specimen surface also play an important role in affecting CO_2_ EC rate. The flow turbulence and availability of sand particles will affect the magnitude of the applied stresses on the specimen surface. Many researchers [6,8,9,16,18] reported that increasing the impingement velocity will increase the flow turbulence and sand loading (in the case of available sand particles). Therefore, higher flow turbulence and sand loading induced by impingement velocity (16 m/s) and availability of sand particles led to much stronger stresses, which ultimately increased the CO_2_ EC as shown in this work.

It is reported by some researchers that the impingement angle and presence of solid particles affect the corrosion product’s removal in terms of the available surface width and depth [15,16]. It was found that at low impingement angles, the removal of corrosion products was quite wider; however, the penetration induced by solid particles was much shallower as compared to high impingement angles. At direct impingement (90°), only surface indentation will occur without significant removal of corrosion products. However, at impingement angle of 45°, there was a balance between normal and shear stresses and that will result in deeper and wider removal of corrosion products than direct impingement (90°). Therefore, the results shown in Figure 11 exhibited the highest CO_2_ EC rate for the specimens tested in the presence of sand, at pH 4.5, an impingement velocity of 16 m/s, and an impingement angle of 45°.

## 4. Conclusions

A detailed investigation was conducted to evaluate the CO_2_ erosion–corrosion performance of API 5L X65 carbon steel under different experimental conditions. The results can be concluded as follows:

- The CO_2_ erosion–corrosion rate decreased with an increase in solution pH;
- Two layers of corrosion scales were observed on the top of the steel surface with different thicknesses and adherent properties at different pH values;
- Cementite (Fe_3_C) and magnetite (Fe_3_O_4_) were present in the corrosion scales formed on specimen surfaces;
- The highest CO_2_ erosion–corrosion was observed at an impingement angle of 45° due to the balance between normal and shear stresses which resulted in deeper erosion than that observed at an impingement angle of 15°;
- The CO_2_ erosion–corrosion rate increased with an increase in impingement velocity due to the increased loading of corrosive spices and higher stresses induced by turbulence flow;
- All specimens tested in the presence of sand particles exhibited much higher CO_2_ EC rates than those tested without sand due to the erosion effect of impinging sand particles.

## Figures and Tables

**Table 1:** Summary of visual observations for all tested specimens.

Effect	without Sand	with Sand
Coloring	Dark coloring was observed, almost in all images	Dark coloring was observed only for pH 5.0 and 5.5. Coloring intensity ↑ as V ↑.
Velocity Effect Scars	Number of scars ↑ as V ↑.	Number of scars ↓ as V ↑.
Pitting	Higher	Lower
